# Non-Ischemic Pattern of LGE After COVID-19 Correlates More with Severity of Acute Illness than with Long-Term Myocardial Dysfunction

**DOI:** 10.3390/jcm14217477

**Published:** 2025-10-22

**Authors:** Alessandro Pingitore, Filippo Figini, Laura Pederzoli, Patrizia Landi, Luca Bastiani, Claudio Marabotti, Filippo Leonardo

**Affiliations:** 1 Clinical Physiology Institute, Counsil National Research (CNR), 56124 Pisa, Italy; patrizia.landi@cnr.it (P.L.); luca.bastiani@cnr.it (L.B.); c.marabotti@gmail.com (C.M.); 2 Ospedale Pederzoli, 37019 Peschiera del Garda, Italy; filippo.figini@ospedalepederzoli.it (F.F.); lpederzoli@yahoo.it (L.P.); filippo65.leonardo@libero.it (F.L.)

**Keywords:** COVID-19, myocarditis, late gadolinium enhancement, cardiac magnetic resonance

## Abstract

**Background/Objectives**: Myocarditis can occur in patients with coronavirus disease 2019 (COVID-19) as part of the systemic involvement of this infectious syndrome. The persistence of this non-ischemic late gadolinium enhancement (LGE) pattern can be considered an indicator of ongoing myocardial involvement or a sequela of myocarditis. We aimed to assess the effects of LGE on cardiac function and morphology in patients with COVID-19 admitted in intensive care unit for acute respiratory distress syndrome. **Methods**: Fifty patients (age 59 ± 11, female *n* = 15) were enrolled. **Results**: The prevalence of LGE was 33.3%. LGE was present in the lateral wall in all patients except for one, with LGE positivity at the interventricular septum. In general, patients with and without LGE had similar CMR variables values. In one case, LGE was associated with regional wall motion abnormality. The factor associated with LGE was the duration of hospitalization (7.97 ± 3.8 and 12.5 ± 6.7 days in patients without and with LGE, *p* = 0.007). **Conclusions**: LGE non-ischemic pattern was not associated with left ventricular dilatation or dysfunction or remodeling in patients with severe clinical manifestation of COVID-19. LGE is mainly present in patients with more prolonged duration of hospitalization. LGE may represent a residual scar with limited prognostic impact that larger multicenter studies could confirm.

## 1. Introduction

Myocarditis can occur in patients with coronavirus disease 2019 (COVID-19) as part of the systemic involvement of this infectious syndrome [1,2,3,4]. The pathophysiology of myocarditis is complex and multifaceted. It may result from direct viral injury, caused by the entry of virions into cardiomyocytes and endothelial cells via angiotensin-converting enzyme 2 (ACE2) receptors, leading to endothelial dysfunction and cellular damage. Alternatively, it may arise through immune-mediated mechanisms, particularly the cytokine storm triggered by severe infection [5,6]. Several studies have documented cases of acute myocarditis in COVID-19, although the reported prevalence varies widely depending on clinical presentation, patient population, and diagnostic criteria [7,8,9,10]. The cohorts examined in these studies have been highly heterogeneous, including individuals with mild to critical illness, those with minimal symptoms, patients requiring ventilatory support, and individuals presenting with respiratory, neurological, or gastrointestinal manifestations. Populations have also differed with respect to baseline cardiovascular status, encompassing both patients with and without pre-existing heart disease, as well as specific subgroups such as athletes [11,12]. In the context of COVID-19, diagnostic approaches for acute myocarditis have been variable, and include measurements of troponin levels, electrocardiography, cardiac magnetic resonance imaging (CMR), and, in some cases, endomyocardial biopsy. Consequently, the reported prevalence of acute myocarditis has ranged widely, from approximately 1% to as high as 26–31% across studies [10,13,14,15,16]. In a large systematic review and meta-analysis including more than 20 million individuals, COVID-19-positive patients (over 1 million) demonstrated an increased risk of incident myocarditis, with a hazard ratio of 5.16 [17]. A more recent meta-analysis confirmed this association, reporting a hazard ratio of 6.11 for COVID-19-positive individuals [18]. Most studies have focused on the acute phase of myocarditis, with limited data available on long-term outcomes of COVID-19-associated myocarditis [10,13,14]. In the available literature, follow-up assessments of late outcomes have typically been conducted 3 to 6 months after acute illness [10,13,14]. Among the diagnostic tools, cardiac magnetic resonance (CMR) has been extensively used to identify myocardial injury in patients with COVID-19 [19,20]. This imaging modality provides detailed information on cardiac morphology and function, as well as myocardial tissue characterization. Notably, myocarditis is commonly associated with a characteristic pattern of late gadolinium enhancement (LGE), typically involving the subepicardial or mid-myocardial layers, most often in the lateral wall. This non-ischemic LGE pattern differs from the subendocardial pattern typically seen in ischemic injury [21,22]. The persistence of this non-ischemic LGE pattern can be considered an indicator of ongoing myocardial involvement or a sequela of myocarditis [23]. In the present study, we enrolled consecutive patients hospitalized with COVID-19-induced acute respiratory distress syndrome (ARDS), who had increased troponin I circulation levels and required intensive care and ventilatory support. None had a prior history of cardiac disease. Patients demonstrating an ischemic LGE patterns on CMR were excluded. The objectives of this study were to assess the clinical factors associated with the presence of a non-ischemic (subepicardial/intramyocardial) LGE pattern and its impact on cardiac function and morphology. We also investigated the prognostic implications of these findings in the study cohort.

## 2. Materials and Methods

### 2.1. Patient Population

This is a retrospective observational single-center study. We enrolled patients who were hospitalized during the first pandemic wave of SARS-CoV-2. The inclusion criteria were the following: (1) patients with a definite diagnosis of COVID-19; (2) patients between 18 and 80 years old; (3) patients who signed their informed consent; (4) patients without previous cardiovascular diseases; (5) patients without severe systemic disease, such as inflammatory diseases; (6) patients without cancer severely limiting life expectancy; patients without chronic renal insufficiency; (7) patients without CMR contraindications; and (8) patients with elevated circulation concentration of Troponin I. The exclusion criteria were as follows: (1) the presence of arrhythmias interfering with the quality of CMR-acquired images; (2) the presence of an ischemic LGE pattern suggesting previous myocardial infarction; (3) patients with global left ventricular dysfunction; (4) patients who refused injection of a gadolinium contrast agent; and (5) patients treated with COVID-19 vaccines. Thus, a total of 51 hospitalized patients with SARS-CoV-2 (mean age 59.1 ± 11.4, female *n* = 15) were enrolled. None received COVID-19 vaccination before CMR examination. COVID-19 positivity was based on a positive real-time polymerase chain reaction (RT-PCR) either on a nasopharyngeal or bronchoalveolar swab. A combined endpoint, involving cardiac death, major arrhythmic events, recurrent episodes of acute myocarditis, and any hospitalization of cardiovascular cause, was used for assessing cardiovascular outcome. This study involving humans was approved by Comitato Etico per la Sperimentazione Clinica (CESC) delle province di Verona e Rovigo (Prog. 3227CESC), approval date 14 June 2021. Informed consent for participation was obtained from all subjects involved in this study. The participants provided their written informed consent to participate in this study. All procedures performed in this study were in accordance with the ethical standards of the institutional and/or national research committee and with the 1964 Helsinki declaration and its later amendments or comparable ethical standards. Informed consent for participation was obtained from all subjects involved in this study.

### 2.2. CMR Protocol and Analysis

CMR was performed on 1.5 Tesla scanners (Magnetom Aera, Siemens Healthcare, Erlangen, Germany) with dedicated cardiac phased array coil. Study protocol included functional evaluation with short axis cine images, acquired from the mitral plane valve to the left ventricular (LV) apex, and these images were acquired using a steady-state free precession pulse sequence with the following parameters: 30 phases, slice thickness 8 mm, no gap, views per segment 8, FOV 35–40 cm, phase FOV 1, matrix 224 × 224, reconstruction matrix 256 × 256, 45° flip angle, and a TR/TE near to 2. Cine images with the same parameters were also acquired in 2-, 3- and 4-chamber views. According to the protocols recommended by the Society for Cardiovascular Magnetic Resonance, we acquired the T1 mapping sequence using a Modified Look-Locker Inversion Recovery (MOLLI) method with a 3 (3) 3 (3) 5 protocol. A 3 (3) 3 (3) 5 scheme indicates that there are a total of 3 inversions. First, 3 images are acquired after the first inversion, and this is followed by a waiting period of 3 RR intervals; then, 3 images are acquired followed by another 3 RR waiting period. Finally, a third inversion, after which 5 images are acquired, for a total of 11 images over 17 heartbeats [24]. T2 mapping used single-shot T2-prepared images acquired at multiple echo times (TE) [25]. Therefore, we obtained three parallel short-axis slices, including the base, mid-cavity, and apex of the left ventricle, at the same cardiac phase (end diastole) both with T1 and T2 mapping. LGE images were acquired 10 min after the administration of Gd-DTPA with a dosage of 0.2 mmol/kg in short-axis views. An inversion recovery T1-weighted gradient-echo (GRE) sequence was used with the following parameters: field of view 35–40 mm, slice thickness 8 mm, no gap between each slice, repetition time 3–5 msec, echo time 1–3, a flip angle of 25°, matrix 224 × 224, reconstruction matrix 256 × 256. The appropriate inversion time was set to null for normal myocardium using a TI-scout. All CMR studies were analyzed offline using a workstation with dedicated CMR software (*syngo*.MR Cardio Engine), with consensus among 2 experienced observers who were blinded to the clinical presentation results. Left ventricular mass was measured by the analysis of the cine short-axis images. The endocardial and epicardial contours of LV myocardium were manually traced in the end-diastolic and the end-systolic phases. End-diastolic volume index, end-systolic volume index, mass, and mass index were measured as previously described [26]. Maximal LV end diastolic wall thickness was measured as previously described. LGE positivity criterion for this study consisted of the presence of subepicardial or mid-wall LGE, corresponding to a non-ischemic pattern of LGE [27]. In the presence of subendocardial/transmural LGE, corresponding to an ischemic LGE pattern, patients were excluded from this study, according to the exclusion criteria. LGE at the right ventricular insertion was not considered as an LGE criterion of positivity.

### 2.3. Statistical Analysis

Continuous variables were reported as the mean ± SD and categoric variables were expressed as absolute numbers (*n*) or percentages (%). Comparisons between the groups were carried out using Student’s t-test for continuous data and the chi-square test (or Fisher test) for categoric variables. All analyses were performed using the IBM Statistical Package for Social Sciences (SPSS, version 23, Chicago 2013), and a two-sided *p*-value < 0.05 was considered statistically significant.

## 3. Results

### 3.1. Patient Clinical Characteristics

The clinical characteristics of all COVID-19 patients are reassumed in Table 1. Hospitalized patients were admitted to hospital for dyspnea at rest and for this reason were treated with O_2_ support. In general, the mean hospital stay was 9 ± 5 days. As shown in Table 1, there were no clinical differences in hospitalized patients with and without LGE, with the exception of hospital stay of duration concerning symptoms that were significantly higher in patients with than those without LGE. In the follow-up (25 ± 5 months), which started after the acute phase of the COVID-19 infection, there was a major cardiac event consisting of the occurrence of ventricular tachycardia requiring cardiac defibrillator implantation.

### 3.2. CMR Variables

**Figure 1:**
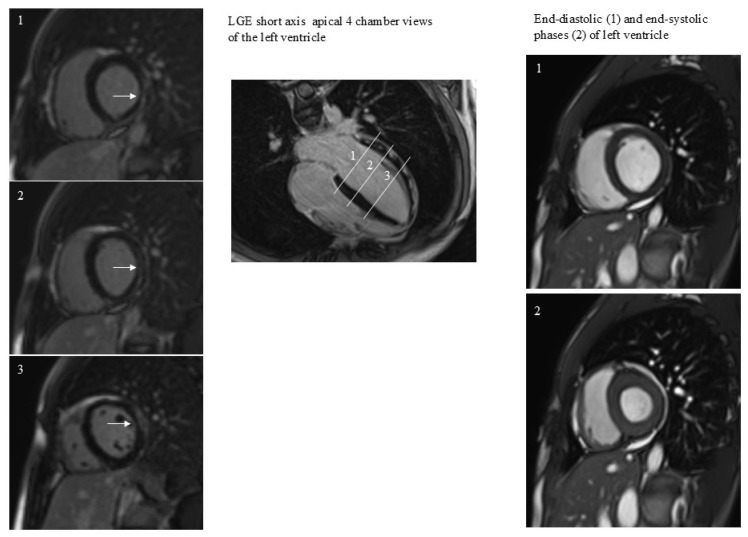
A case of subepicardial and intramyocardial LGE (arrows). LGE persistence was not associated with dilatation or regional dysfunction of the left ventricle. The 3 white lines correspond to the 3 short axes planes on the left.

**Figure 2:**
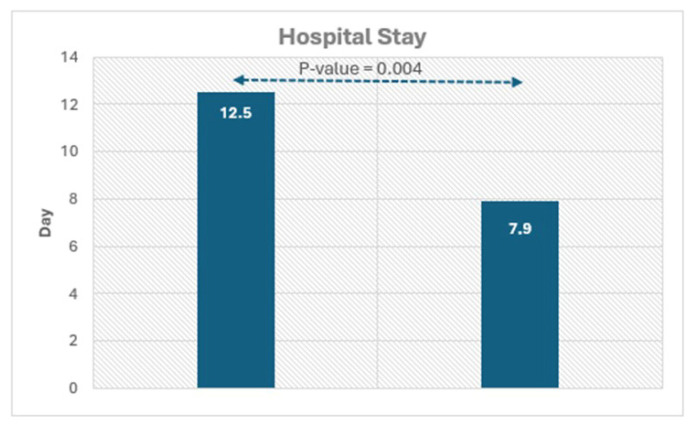
Histograms showing the significant difference in hospital duration in patients with and without LGE.

All patients performed CMR examination without any kind of complication. The time interval between COVID-19 diagnosis and CMR was 409 ± 26 days. The prevalence of positive LGE was 33% (17/51). LGE was present in the lateral wall in all patients except for one patient, with LGE positivity at the level of the interventricular septum (Figure 1). In this patient LGE was associated with abnormal regional wall motion (hypokinesia). In general, patients, both with and without positive LGE, had similar CMR variable values (Table 1). The factor associated with LGE was the duration of hospitalization (Figure 2).

## 4. Discussion

This study primarily investigated the presence of non-ischemic LGE pattern on CMR in patients positive for COVID-19, hospitalized for ARDS, requiring intensive care and ventilatory support during the first pandemic wave, without previous cardiac or systemic diseases. The findings showed that non-ischemic LGE, considered a potential marker of post-myocarditis sequelae, was not associated with regional or global impairment of cardiac function or ventricular remodeling. Residual pericardial effusion was frequently documented but lacked hemodynamic significance. Interestingly, patients with LGE positivity had a longer hospitalization period and higher duration of symptoms. These findings should be interpreted in the context of the study population: patients without prior cardiovascular events or systemic comorbidities, who presented with a non-ischemic pattern of LGE potentially inferring the clinical course of myocarditis. The results of this study are consistent with those of previous ones showing the low clinical impact of myocarditis, detected with LGE when localized in the lateral segments. Previous studies, involving patients with non-COVID-19 myocarditis detected with CMR, assessed the prognostic impact of LGE patterns of myocarditis, including its presence, LV segmental location, the extent, and the intramyocardial site of LGE. In the ITAMY study, Aquaro et al. reported that mid-wall LGE localized in the anteroseptal LV segment was associated with a worse prognosis among patients with acute myocarditis and preserved left ventricular ejection fraction (LVEF) [28]. Similarly, Grani et al. found that the coexistence of LGE and LV dysfunction predicted adverse outcomes, with septal and mid-wall LGE patterns showing the strongest associations with major cardiac events [29]. A meta-analysis by Georgiopoulos G et al., including 2328 patients from 11 independent cohorts, confirmed that the presence of LGE and the anteroseptal location, but not LGE extent, were associated with an increased risk of the combined end point of all-cause mortality and major adverse cardiac events [30]. It is also interesting to consider the results of Mahrholdt et al., who showed that the pattern of myocardial damage was related to the type of virus [31]. Specifically, patients with parvovirus B19 (PVB19)-induced myocarditis exhibited subepicardial LGE in the lateral wall, whereas those with human herpesvirus 6 (HHV6)-induced myocarditis showed predominantly intramural anteroseptal LGE, similar to cases of combined PVB19/HHV6 infection [31]. Notably, clinical outcomes differed according to etiology: patients with HHV6 or dual HHV6/PVB19 myocarditis more often developed heart failure and progressed toward chronic cardiac dysfunction. It is obvious that the clinical impact of myocarditis is influenced by LV morphology and function. As reported by Filippetti et al., the presence of LV remodeling at mid-term follow-up carried prognostic significance in patients with myocarditis [32]. In the present study, myocarditis predominantly involved the lateral wall in all but one patient, who exhibited anteroseptal involvement associated with regional wall motion abnormality. However, none of the patients had LV remodeling. In accordance with the above-mentioned studies, our findings suggest that the myocardial distribution of LGE may depend on the causative pathogen, which in turn influences mid-term cardiac outcomes. Further investigations are warranted to confirm this hypothesis.

Regarding previous CMR studies on COVID-19-induced myocarditis, our study differs from most of them, since we performed CMR after almost one year from the onset of COVID-19 infection, in a condition of presumably full clinical and healing process stability [10,13,14,33,34,35,36]. This timing explains the absence of myocardial edema and indicates that the observed LGE represents permanent, irreversible myocardial injury. Moreover, we exclusively included patients with a non-ischemic pattern of LGE, that is, a subepicardial/intramyocardial LGE pattern, thereby excluding patients with ischemic injury. This methodological distinction contrasts with studies such as those by Vidula et al., who enrolled patients with prior myocardial infarction, known coronary artery disease, or pre-existing cardiomyopathies, among whom 6.7% demonstrated ischemic injury patterns [35]. Similarly, in the study by Artico et al., patients with prior cardiac events who underwent CMR within 28 days of discharge showed a high incidence of ischemic LGE [36]. At mid-to-long term follow-up in our population, only one patient had a major cardiac event at follow-up, consisting of ventricular tachycardia. It is obvious that our study does not have the strength to define the prognostic impact of a non-ischemic pattern of LGE in patients positive for COVID-19. However, in the study of Yar et al., patients at follow-up did not have higher rates of dyspnea, chest pain, arrhythmias, or syncope, in comparison to those without LGE [37]. Likewise, Shiwani H. et al. recently reported that myocardial injury in hospitalized COVID-19 patients was non-progressive, with a low incidence of major cardiovascular events at mid-term follow-up and no deterioration in LV function [7]. Conversely, a high incidence of acute cardiac events has been documented during the initial hospitalization phase of COVID-19 infection [8,38]. In that setting, the adverse prognosis of myocardial injury is largely attributable to multisystem involvement and the severity of critical illness [38,39,40]. Therefore, the clinical implication of our study is that a non-ischemic LGE pattern, predominantly localized in the lateral wall, appears to have limited long-term cardiac significance in a highly selected cohort of patients who survived severe COVID-19 infection without prior cardiovascular or systemic disease.

### Limitation of the Study

We cannot define the true prevalence of COVID-19-induced non-ischemic LGE pattern on CMR; however, this was not the primary objective of our study. This is because we selected patients with high values of troponin during hospitalization. In previous studies, the prevalence of myocarditis varied according to the kind of patients enrolled, and also according to the definition of myocardial injury. Moreover, CMR in our study was performed after the acute phase of COVID-19 infection, which may have led to underestimation by missing transient cases that resolved without structural sequelae. Although in our population we showed a limited prognostic impact of COVID-19-induced non-ischemic LGE pattern, this conclusion must be interpreted with caution given the small sample size and the potential for selection bias. Larger studies are required to confirm these findings. Furthermore, elevated troponin levels in patients with respiratory distress may reflect right ventricular strain or failure rather than direct myocardial inflammation [41,42]. Nevertheless, the presence of subepicardial or intramyocardial LGE remains an established CMR marker of prior myocarditis and has been consistently used in previous studies to identify COVID-19-related myocardial injury [16,23,37].

## 5. Conclusions

In this study, the presence of LGE, interpreted as a potential indicator of post-inflammatory myocardial injury induced by COVID-19, was not associated with dilation, global or regional dysfunction, or remodeling of the left ventricle. Conversely, LGE correlated with indicators of disease severity, such as longer hospitalization duration or duration of symptoms. These findings suggest that LGE may represent residual myocardial scarring with limited long-term prognostic significance. Future large, multicenter studies are warranted to confirm this hypothesis and to better characterize the clinical implications of non-ischemic LGE in survivors of severe COVID-19 infection.

## Figures and Tables

**Table 1:** All patients clinical and CMR characteristics.

Variables	Positive LGE (*n* = 17)	Negative LGE (*n* = 34)	*p* Value
Age	57.8 ± 13.3	52.27 ± 16.3	0.124
Sex (%)	68% (M) 32% (F)	57.8% (M) 42.2% (F)	0.356
Hospital Stay	11.21 ± 7.5	3.61 ± 4.7	<0.001
Duration of Symptoms	8.45 ± 4.9	4.69 ± 4.2	<0.001
Hypertension (%)	44.4%	35.9%	0.500
Diabetes (%)	27.8%	9.0%	0.03 *
Dyslipidemia (%)	55.6%	33.8%	0.086
Smoker or ex smoker	28.6%	27.8%	0.899
Cough (%)	80.0%	43.3%	0.001 *
Syncope (%)	12.0%	2.2%	0.034
Fever (%)	96.0%	61.1%	<0.001
Nausea (%)	12.0%	16.7%	0.570
Ageusia/Anosmia (%)	8.0%	18.9%	0.195
Diarrhea (%)	8.0%	5.6%	0.651
Asthenia	40.0%	33.3%	0.536
Myalgia (%)	48.0%	43.3%	0.678
LV EDV (mL/m^2^)	72.52 ± 13.2	72.18 ± 15.9	0.922
LV ESV (mL/m^2^)	29.28 ± 9.1	26.49 ± 8.5	0.155
LV Mass (gr/m^2^)	72.0 ± 10.3	68.8 ± 11.9	0.230
LV SV (mL/m^2^)	44.5 ± 6.2	46.6 ± 9.0	0.271
LV CO (L/min)	5.5 ± 1.2	6.12 ± 6.54	0.348
LV EF (%)	62.28 ± 7	65.27 ± 5	<0.024
WMSI	1.02 ± 0.13	1 ± 0	<0.041
RV EDV (mL/m^2^)	69.8 ± 13.2	70.7 ± 17.2	0.820
RV ESV (mL/m^2^)	25.8 ± 8.7	25.7 ± 10.9	0.971
RV SV (mL/m^2^)	47.4 ± 13.9	46.2 ± 12.1	0.682
RV CO (L/min)	5.46 ± 1.43	5.89 ± 1.38	0.177
RV EF (%)	63.5 ± 7	65.9 ± 6	0.05
R/L EVD relationship	0.97 ± 0.13	0.97 ± 0.15	0.988
Native T1 (msec)	992 ± 54.4	1006 ± 37.6	0.140
ECV (%)	25.5 ± 2.58	25.9 ± 2.43	0.480
Native T2 (msec)	49.6 ± 2.7	50.4 ± 3.4	0.965
Pericardial effusion	12 (48%)	22 (24.4%)	0.022

LV EDV = Left Ventricular End-Diastolic Volume; LV ESV = LV End-Systolic Volume; LV SV = LV Stroke Volume; LV CO = LV Cardiac Output; LV EF = LV Ejection Fraction; WMSI = Wall Motion Score Index; RV = Right Ventricular; R/L EDV = Right/Left EDV relationship; ECV = extracellular volume; * Fisher Exact Test.

## Data Availability

The data are available. Please request them via the corresponding author.

## Abbreviations

The following abbreviations are used in this manuscript:

CMR	cardiac magnetic resonance
LGE	late gadolinium enhancement
ARDS	acute respiratory distress syndrome
LV	left ventricle
